# (3,6-Dimeth­oxy­naphthalen-2-yl)(naphthalen-2-yl)methanone

**DOI:** 10.1107/S1600536812034186

**Published:** 2012-08-08

**Authors:** Takehiro Tsumuki, Shun Murohashi, Atsushi Nagasawa, Akiko Okamoto, Noriyuki Yonezawa

**Affiliations:** a Department of Organic and Polymer Materials Chemistry, Tokyo University of Agriculture & Technology, 2-24-16 Naka-machi, Koganei, Tokyo 184-8588, Japan

## Abstract

In the title compound, C_23_H_18_O_3_, the dihedral angle between the two naphthalene ring systems is 78.02 (3)°. The bridging carbonyl C—C(=O)—C plane makes a dihedral angle of 70.56 (5)° with the naphthalene ring system in the 2,7-dimeth­oxy­naphthalene moiety and a dihedral angle of 11.53 (5)° with the naphthalene ring system in the naphthoyl group. In the crystal, adjacent mol­ecules are linked *via* C—H⋯π inter­actions, forming chains along [010].

## Related literature

For electrophilic aromatic aroylation of naphthalene derivatives, see: Okamoto & Yonezawa (2009 ▶); Okamoto *et al.* (2011 ▶). For the structures of closely related compounds, see: Kato *et al.* (2010 ▶, 2011 ▶); Nakaema *et al.* (2008 ▶); Tsumuki *et al.* (2011 ▶, 2012 ▶); Watanabe *et al.* (2010 ▶).
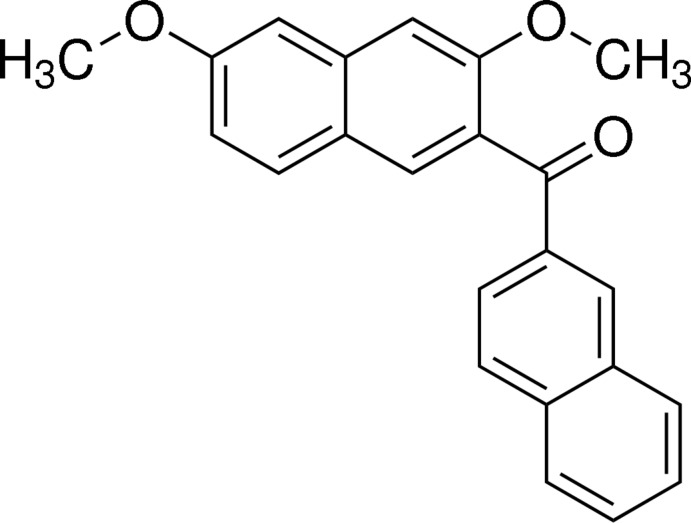


## Experimental

### 

#### Crystal data

C_23_H_18_O_3_
*M* *_r_* = 342.37
Monoclinic, 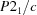

*a* = 13.4683 (9) Å
*b* = 8.9062 (5) Å
*c* = 14.7110 (8) Å
β = 105.646 (2)°
*V* = 1699.23 (17) Å^3^
*Z* = 4
Mo *K*α radiation
μ = 0.09 mm^−1^
*T* = 193 K
0.60 × 0.30 × 0.20 mm

#### Data collection

Rigaku R-AXIS RAPID diffractometer
Absorption correction: numerical (*NUMABS*; Higashi, 1999 ▶) *T*_min_ = 0.941, *T*_max_ = 0.983
26536 measured reflections
3863 independent reflections
3436 reflections with *I* > 2σ(*I*)
*R*_int_ = 0.017

#### Refinement

*R*[*F* ^2^ > 2σ(*F* ^2^)] = 0.038
*wR*(*F* ^2^) = 0.109
*S* = 1.06
3863 reflections
238 parameters
H-atom parameters constrained
Δρ_max_ = 0.31 e Å^−3^
Δρ_min_ = −0.18 e Å^−3^

### 

Data collection: *PROCESS-AUTO* (Rigaku, 1998 ▶); cell refinement: *PROCESS-AUTO*; data reduction: *PROCESS-AUTO*; program(s) used to solve structure: *SIR2004* (Burla *et al.*, 2005 ▶); program(s) used to refine structure: *SHELXL97* (Sheldrick, 2008 ▶); molecular graphics: *ORTEPIII* (Burnett & Johnson, 1996 ▶); software used to prepare material for publication: *SHELXL97*.

## Supplementary Material

Crystal structure: contains datablock(s) I, global. DOI: 10.1107/S1600536812034186/su2487sup1.cif

Structure factors: contains datablock(s) I. DOI: 10.1107/S1600536812034186/su2487Isup2.hkl

Supplementary material file. DOI: 10.1107/S1600536812034186/su2487Isup3.cml

Additional supplementary materials:  crystallographic information; 3D view; checkCIF report

## Figures and Tables

**Table 1:** Hydrogen-bond geometry (Å, °) *Cg*1 is the centroid of the C14–C19 ring.

*D*—H⋯*A*	*D*—H	H⋯*A*	*D*⋯*A*	*D*—H⋯*A*
C22—H22*A*⋯*Cg*4^i^	0.98	2.80	3.5470 (12)	133

Symmetry code: (i) 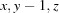.

**Fig. 1.**
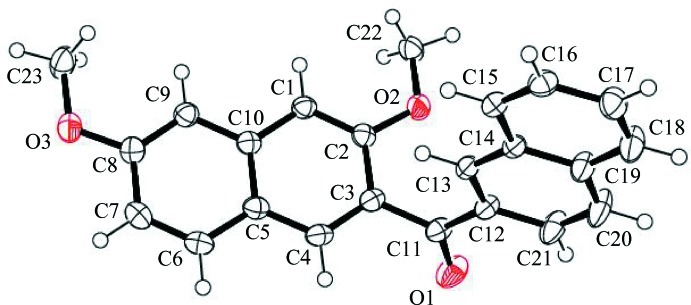
The molecular structure of the title molecule, with the atom labeling. Displacement ellipsoids are drawn at the 50% probability level.

**Fig. 2.**
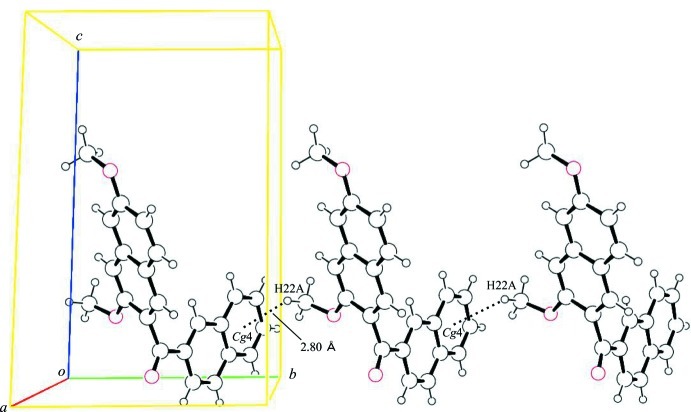
A partial view along the a axis of the crystal packing of the title compound, showing the C—H···π interaction as dashed lines (see Table for details).

## supplementary crystallographic information

### Comment

In the course of our studies on selective electrophilic aromatic aroylation of 2,7-dimethoxynaphthalene, *peri*-aroylnaphthalene compounds have proved to be formed regioselectively with the aid of suitable acidic mediator (Okamoto & Yonezawa, 2009; Okamoto *et al.*, 2011). We have reported the structures of 1,8-dibenzoylnaphthalene analogues such as 1,8-dibenzoyl-2,7-dimethoxynaphthalene (Nakaema *et al.*, 2008). The benzoyl groups at the 1,8-positions of the naphthalene rings in these compounds are bonded in a nearly perpendicular manner and orient in opposite directions. The 1-monobenzoylnaphthalene analogues, such as (2,7-dimethoxynaphthalen-1-yl)(phenyl)methanone (Kato *et al.*, 2010), were also revealed to have essentially the same non-coplanar structure as observed for 1,8-dibenzoylated naphthalene analogues. The corresponding *β*-isomers of 3-monobenzoylated naphthalene analogues such as (3,6-dimethoxynaphthalen-2-yl)(phenyl)methanone (Kato, *et al.*, 2011) and (4-fluorophenyl)(3,6-dimethoxy-2-naphthyl)methanone (Watanabe *et al.*, 2010). In the 3-monobenzoylated naphthalene analogues, which are generally regarded to be thermodynamically more stable than the corresponding 1-positioned isomeric molecules, the aroyl groups are connected to the naphthalene rings in a moderately twisted fashion.

Recently, a series of the corresponding naphthoylated naphthalene homologues to the benzoylated naphthalenes have been reported, such as [2,7-dimethoxy-8-(2-naphthoyl)naphthalen-1-yl](naphthalen-2-yl)methanone (Tsumuki *et al.*, 2011) and 1-(2-naphthoyl)-2,7-dimethoxynaphthalene (Tsumuki *et al.*, 2012). As a part of our ongoing studies on the synthesis and structure of these homologous molecules, the crystal structure analysis of the title compound, a 2,7-dimethoxynaphthalene substituted at the 3-position by a 2-naphthoyl group, is reported on herein.

The molecular structure of the title molecule is illustrated in Fig. 1. The interplanar angle between the two naphthalene rings (C1—C10 and C12—C21) is 78.02 (3)°. The dihedral angle between the bridging carbonyl plane (O1—C3—C11—C12) and the naphthalene ring of the 2,7-demethoxynaphthalene moiety (C1—C10) is larger than that between the bridging carbonyl plane (O1—C3—C11—C12) and naphthalene ring of the naphthoyl group (C12—C21) [70.56 (5)° *versus*. 11.53 (5)°; torsion angle C2—C3—C11—O1 = -110.65 (13)° *versus*. torsion angle O1—C11—C12—C13 = -167.08 (11)°]

In the crystal, neighbouring molecules are linked by C—H···π interactions along the *b* axis (Table 1 and Fig. 2).

### Experimental

The title compound was prepared by treatment of a mixture of 2,7-dimethoxynaphthalene (188 mg, 1 mmol) and 2-naphthoic acid (189 mg, 1.1 mmol) with phosphorus pentoxide—methanesulfonic acid mixture (P_2_O_5_—MsOH [1/10 *w*/*w*] 2.2 ml). After the reaction mixture had been stirred at 333 K for 6 h, the mixture was poured into ice-cold water and extracted with CHCl_3_ (3 × 10 ml). The combined extracts were washed with 2 *M* aqueous NaOH (3 × 15 ml) followed by washing with brine ( 3 × 15 ml). The organic layer thus obtained was dried over anhydrous MgSO_4_. The solvent was removed under reduced pressure to give a cake (yield 349 mg, quant.). The crude product was purified by flush silica gel chromatography (eluent: toluene; isolated yield 28%). Colourless platelet single crystals suitable for X-ray diffraction were obtained by crystallization from chloroform. Spectroscopic data for the title compound are given in the archived CIF.

### Refinement

All the H atoms could be located in a difference Fourier map. In the final cycles of refinement they were included in calculated positions and treated as riding atoms: C—H = 0.95 (aromatic) and 0.98 (methyl) Å, with *U*_iso_(H) = 1.2*U*_eq_(C).

### Crystal data

**Table:**

C_23_H_18_O_3_	*F*(000) = 720
*M**_r_* = 342.37	*D*_x_ = 1.338 Mg m^−^^3^
Monoclinic, *P*2_1_/*c*	Melting point = 444.0–445.0 K
Hall symbol: -P 2ybc	Mo *K*α radiation, λ = 0.71075 Å
*a* = 13.4683 (9) Å	Cell parameters from 20190 reflections
*b* = 8.9062 (5) Å	θ = 3.1–27.4°
*c* = 14.7110 (8) Å	µ = 0.09 mm^−^^1^
β = 105.646 (2)°	*T* = 193 K
*V* = 1699.23 (17) Å^3^	Block, colourless
*Z* = 4	0.60 × 0.30 × 0.20 mm

### Data collection

**Table:**

Rigaku R-AXIS RAPID diffractometer	3863 independent reflections
Radiation source: rotating anode	3436 reflections with *I* > 2σ(*I*)
Graphite monochromator	*R*_int_ = 0.017
Detector resolution: 10.00 pixels mm^-1^	θ_max_ = 27.5°, θ_min_ = 3.1°
ω scans	*h* = −17→17
Absorption correction: numerical (*NUMABS*; Higashi, 1999)	*k* = −11→11
*T*_min_ = 0.941, *T*_max_ = 0.983	*l* = −19→18
26536 measured reflections	

### Refinement

**Table:**

Refinement on *F*^2^	Secondary atom site location: difference Fourier map
Least-squares matrix: full	Hydrogen site location: inferred from neighbouring sites
*R*[*F*^2^ > 2σ(*F*^2^)] = 0.038	H-atom parameters constrained
*wR*(*F*^2^) = 0.109	*w* = 1/[σ^2^(*F*_o_^2^) + (0.0609*P*)^2^ + 0.3821*P*] where *P* = (*F*_o_^2^ + 2*F*_c_^2^)/3
*S* = 1.06	(Δ/σ)_max_ < 0.001
3863 reflections	Δρ_max_ = 0.31 e Å^−^^3^
238 parameters	Δρ_min_ = −0.18 e Å^−^^3^
0 restraints	Extinction correction: *SHELXL97* (Sheldrick, 2008), Fc^*^=kFc[1+0.001xFc^2^λ^3^/sin(2θ)]^-1/4^
Primary atom site location: structure-invariant direct methods	Extinction coefficient: 0.0154 (19)

### Special details

**Table:**

Experimental. Spectroscopic data for the title compound: ^1^H NMR δ (300 MHz, CDCl_3_): 3.83 (3*H*, s), 3.96 (3*H*, s), 7.07 (1*H*, dd, J = 2.4, 9.0 Hz), 7.14 (1*H*, d, J = 2.4 Hz), 7.18 (1*H*, s), 7.51 (1*H*, dt, J = 1.2, 7.5 Hz), 7.60 (1*H*, dt, J = 1.2, 7.5 Hz), 7.71 (1*H*, d, J = 9.0 Hz), 7.84–7.92 (4*H*, m), 8.02 (1*H*, dd, J = 1.2, 9.0 Hz), 8.26 (1*H*,d, J = 1.2 Hz) p.p.m. ^13^C NMR δ (125 MHz, CDCl_3_): 195.97, 159.32, 155.89, 137.11, 135.55, 135.46, 132.39, 132.19, 130.03, 130.01, 129.59, 128.35, 128.05, 128.02, 127.73, 126.52, 125.17, 123.17, 117.02, 105.43, 105.04, 55.56, 55.32 p.p.m. IR (KBr, cm^-1^): 1666, 1630, 1459, 1226, 1191 HRMS (*m/z*): [*M*+H]^+^ calcd. for C_23_H_19_O_3_, 343.1334, found, 343.1344.
Geometry. All e.s.d.'s (except the e.s.d. in the dihedral angle between two l.s. planes) are estimated using the full covariance matrix. The cell e.s.d.'s are taken into account individually in the estimation of e.s.d.'s in distances, angles and torsion angles; correlations between e.s.d.'s in cell parameters are only used when they are defined by crystal symmetry. An approximate (isotropic) treatment of cell e.s.d.'s is used for estimating e.s.d.'s involving l.s. planes.

### Fractional atomic coordinates and isotropic or equivalent isotropic displacement parameters (Å^2^)

**Table:**

	*x*	*y*	*z*	*U*_iso_*/*U*_eq_	
O1	0.31909 (7)	0.44270 (11)	0.02517 (6)	0.0449 (2)	
O2	0.21312 (6)	0.25562 (9)	0.18816 (5)	0.03164 (19)	
O3	0.66735 (6)	0.30077 (10)	0.59573 (6)	0.0396 (2)	
C1	0.35816 (8)	0.26610 (11)	0.32895 (7)	0.0256 (2)	
H1	0.3284	0.1931	0.3606	0.031*	
C2	0.30615 (7)	0.31204 (11)	0.23965 (7)	0.0248 (2)	
C3	0.34880 (8)	0.42318 (11)	0.19167 (7)	0.0252 (2)	
C4	0.44353 (8)	0.48307 (12)	0.23457 (7)	0.0282 (2)	
H4	0.4720	0.5567	0.2022	0.034*	
C5	0.50006 (8)	0.43750 (11)	0.32631 (7)	0.0270 (2)	
C6	0.59859 (9)	0.49671 (14)	0.37265 (8)	0.0363 (3)	
H6	0.6283	0.5713	0.3420	0.044*	
C7	0.65131 (9)	0.44839 (15)	0.46046 (9)	0.0390 (3)	
H7	0.7175	0.4886	0.4900	0.047*	
C8	0.60763 (8)	0.33873 (13)	0.50744 (7)	0.0311 (2)	
C9	0.51225 (8)	0.27940 (12)	0.46576 (7)	0.0278 (2)	
H9	0.4834	0.2062	0.4981	0.033*	
C10	0.45652 (7)	0.32767 (11)	0.37402 (7)	0.0244 (2)	
C11	0.28960 (8)	0.47414 (12)	0.09415 (7)	0.0279 (2)	
C12	0.19693 (8)	0.56988 (11)	0.08637 (7)	0.0263 (2)	
C13	0.17608 (7)	0.62890 (11)	0.16571 (6)	0.0236 (2)	
H13	0.2198	0.6049	0.2262	0.028*	
C14	0.09043 (7)	0.72487 (11)	0.15864 (7)	0.0233 (2)	
C15	0.06908 (8)	0.78952 (11)	0.23953 (7)	0.0260 (2)	
H15	0.1131	0.7686	0.3004	0.031*	
C16	−0.01430 (8)	0.88170 (12)	0.23057 (7)	0.0300 (2)	
H16	−0.0272	0.9255	0.2851	0.036*	
C17	−0.08113 (9)	0.91196 (14)	0.14072 (8)	0.0358 (3)	
H17	−0.1393	0.9751	0.1352	0.043*	
C18	−0.06276 (9)	0.85107 (15)	0.06142 (8)	0.0408 (3)	
H18	−0.1086	0.8719	0.0013	0.049*	
C19	0.02400 (9)	0.75705 (13)	0.06789 (7)	0.0318 (2)	
C20	0.04710 (10)	0.69372 (16)	−0.01265 (8)	0.0444 (3)	
H20	0.0031	0.7144	−0.0736	0.053*	
C21	0.13096 (10)	0.60409 (15)	−0.00430 (7)	0.0390 (3)	
H21	0.1455	0.5642	−0.0592	0.047*	
C22	0.17610 (8)	0.12457 (12)	0.22435 (8)	0.0334 (2)	
H22A	0.1108	0.0929	0.1808	0.040*	
H22B	0.1652	0.1471	0.2861	0.040*	
H22C	0.2269	0.0437	0.2310	0.040*	
C23	0.62408 (10)	0.19186 (14)	0.64517 (9)	0.0407 (3)	
H23A	0.6707	0.1764	0.7083	0.049*	
H23B	0.6147	0.0968	0.6103	0.049*	
H23C	0.5572	0.2276	0.6508	0.049*	

### Atomic displacement parameters (Å^2^)

**Table:**

	*U*^11^	*U*^22^	*U*^33^	*U*^12^	*U*^13^	*U*^23^
O1	0.0548 (5)	0.0553 (5)	0.0303 (4)	0.0209 (4)	0.0210 (4)	0.0045 (4)
O2	0.0279 (4)	0.0306 (4)	0.0328 (4)	−0.0027 (3)	0.0019 (3)	0.0078 (3)
O3	0.0329 (4)	0.0456 (5)	0.0342 (4)	−0.0046 (4)	−0.0015 (3)	0.0070 (4)
C1	0.0263 (5)	0.0232 (4)	0.0286 (5)	0.0005 (4)	0.0095 (4)	0.0039 (4)
C2	0.0232 (5)	0.0233 (5)	0.0286 (5)	0.0027 (4)	0.0082 (4)	0.0008 (4)
C3	0.0278 (5)	0.0241 (5)	0.0262 (5)	0.0057 (4)	0.0114 (4)	0.0029 (4)
C4	0.0308 (5)	0.0265 (5)	0.0310 (5)	0.0010 (4)	0.0146 (4)	0.0051 (4)
C5	0.0264 (5)	0.0268 (5)	0.0302 (5)	0.0006 (4)	0.0117 (4)	0.0014 (4)
C6	0.0312 (5)	0.0400 (6)	0.0391 (6)	−0.0085 (5)	0.0120 (5)	0.0065 (5)
C7	0.0279 (5)	0.0464 (7)	0.0404 (6)	−0.0097 (5)	0.0051 (5)	0.0026 (5)
C8	0.0284 (5)	0.0337 (5)	0.0297 (5)	0.0013 (4)	0.0054 (4)	0.0017 (4)
C9	0.0282 (5)	0.0264 (5)	0.0289 (5)	0.0010 (4)	0.0082 (4)	0.0033 (4)
C10	0.0246 (5)	0.0226 (4)	0.0273 (5)	0.0029 (4)	0.0093 (4)	0.0007 (4)
C11	0.0337 (5)	0.0268 (5)	0.0256 (5)	0.0034 (4)	0.0121 (4)	0.0025 (4)
C12	0.0311 (5)	0.0266 (5)	0.0222 (5)	0.0033 (4)	0.0086 (4)	0.0031 (4)
C13	0.0263 (5)	0.0241 (5)	0.0199 (4)	−0.0001 (4)	0.0053 (3)	0.0024 (3)
C14	0.0260 (5)	0.0235 (4)	0.0203 (4)	−0.0009 (4)	0.0061 (4)	0.0013 (3)
C15	0.0300 (5)	0.0269 (5)	0.0215 (4)	0.0002 (4)	0.0075 (4)	0.0010 (4)
C16	0.0343 (5)	0.0304 (5)	0.0280 (5)	0.0021 (4)	0.0131 (4)	−0.0007 (4)
C17	0.0318 (5)	0.0402 (6)	0.0354 (6)	0.0113 (5)	0.0091 (4)	0.0022 (5)
C18	0.0384 (6)	0.0526 (7)	0.0273 (5)	0.0175 (5)	0.0019 (4)	0.0023 (5)
C19	0.0339 (6)	0.0377 (6)	0.0221 (5)	0.0085 (4)	0.0045 (4)	0.0017 (4)
C20	0.0511 (7)	0.0594 (8)	0.0182 (5)	0.0231 (6)	0.0018 (5)	0.0016 (5)
C21	0.0497 (7)	0.0483 (7)	0.0191 (5)	0.0172 (6)	0.0092 (5)	0.0007 (4)
C22	0.0270 (5)	0.0302 (5)	0.0404 (6)	−0.0029 (4)	0.0049 (4)	0.0066 (4)
C23	0.0456 (7)	0.0363 (6)	0.0335 (6)	−0.0015 (5)	−0.0009 (5)	0.0082 (5)

### Geometric parameters (Å, º)

**Table:**

O1—C11	1.2179 (13)	C12—C21	1.4221 (14)
O2—C2	1.3726 (12)	C13—C14	1.4168 (14)
O2—C22	1.4274 (13)	C13—H13	0.9500
O3—C8	1.3729 (13)	C14—C15	1.4195 (13)
O3—C23	1.4276 (15)	C14—C19	1.4215 (14)
C1—C2	1.3739 (14)	C15—C16	1.3684 (14)
C1—C10	1.4219 (14)	C15—H15	0.9500
C1—H1	0.9500	C16—C17	1.4101 (15)
C2—C3	1.4235 (14)	C16—H16	0.9500
C3—C4	1.3698 (15)	C17—C18	1.3683 (16)
C3—C11	1.5109 (14)	C17—H17	0.9500
C4—C5	1.4183 (15)	C18—C19	1.4198 (15)
C4—H4	0.9500	C18—H18	0.9500
C5—C10	1.4195 (14)	C19—C20	1.4204 (15)
C5—C6	1.4198 (15)	C20—C21	1.3613 (17)
C6—C7	1.3651 (17)	C20—H20	0.9500
C6—H6	0.9500	C21—H21	0.9500
C7—C8	1.4131 (16)	C22—H22A	0.9800
C7—H7	0.9500	C22—H22B	0.9800
C8—C9	1.3713 (15)	C22—H22C	0.9800
C9—C10	1.4223 (14)	C23—H23A	0.9800
C9—H9	0.9500	C23—H23B	0.9800
C11—C12	1.4903 (14)	C23—H23C	0.9800
C12—C13	1.3762 (13)		
			
C2—O2—C22	116.88 (8)	C12—C13—H13	119.5
C8—O3—C23	115.71 (9)	C14—C13—H13	119.5
C2—C1—C10	120.12 (9)	C13—C14—C15	121.89 (9)
C2—C1—H1	119.9	C13—C14—C19	118.97 (9)
C10—C1—H1	119.9	C15—C14—C19	119.15 (9)
O2—C2—C1	124.94 (9)	C16—C15—C14	120.61 (9)
O2—C2—C3	114.29 (9)	C16—C15—H15	119.7
C1—C2—C3	120.75 (9)	C14—C15—H15	119.7
C4—C3—C2	119.51 (9)	C15—C16—C17	120.32 (10)
C4—C3—C11	120.52 (9)	C15—C16—H16	119.8
C2—C3—C11	119.97 (9)	C17—C16—H16	119.8
C3—C4—C5	121.41 (9)	C18—C17—C16	120.46 (10)
C3—C4—H4	119.3	C18—C17—H17	119.8
C5—C4—H4	119.3	C16—C17—H17	119.8
C4—C5—C10	118.71 (9)	C17—C18—C19	120.75 (10)
C4—C5—C6	122.88 (10)	C17—C18—H18	119.6
C10—C5—C6	118.41 (9)	C19—C18—H18	119.6
C7—C6—C5	121.17 (10)	C18—C19—C20	122.66 (10)
C7—C6—H6	119.4	C18—C19—C14	118.69 (10)
C5—C6—H6	119.4	C20—C19—C14	118.65 (10)
C6—C7—C8	120.11 (10)	C21—C20—C19	121.38 (10)
C6—C7—H7	119.9	C21—C20—H20	119.3
C8—C7—H7	119.9	C19—C20—H20	119.3
C9—C8—O3	124.56 (10)	C20—C21—C12	120.23 (10)
C9—C8—C7	120.73 (10)	C20—C21—H21	119.9
O3—C8—C7	114.70 (10)	C12—C21—H21	119.9
C8—C9—C10	119.91 (9)	O2—C22—H22A	109.5
C8—C9—H9	120.0	O2—C22—H22B	109.5
C10—C9—H9	120.0	H22A—C22—H22B	109.5
C5—C10—C1	119.49 (9)	O2—C22—H22C	109.5
C5—C10—C9	119.65 (9)	H22A—C22—H22C	109.5
C1—C10—C9	120.85 (9)	H22B—C22—H22C	109.5
O1—C11—C12	121.47 (9)	O3—C23—H23A	109.5
O1—C11—C3	121.00 (10)	O3—C23—H23B	109.5
C12—C11—C3	117.47 (8)	H23A—C23—H23B	109.5
C13—C12—C21	119.66 (9)	O3—C23—H23C	109.5
C13—C12—C11	120.77 (9)	H23A—C23—H23C	109.5
C21—C12—C11	119.54 (9)	H23B—C23—H23C	109.5
C12—C13—C14	121.10 (9)		
			
C22—O2—C2—C1	−10.08 (15)	C4—C3—C11—O1	69.33 (15)
C22—O2—C2—C3	168.17 (9)	C2—C3—C11—O1	−110.65 (12)
C10—C1—C2—O2	177.09 (9)	C4—C3—C11—C12	−107.91 (11)
C10—C1—C2—C3	−1.05 (15)	C2—C3—C11—C12	72.11 (12)
O2—C2—C3—C4	−177.28 (9)	O1—C11—C12—C13	−167.09 (11)
C1—C2—C3—C4	1.06 (15)	C3—C11—C12—C13	10.14 (15)
O2—C2—C3—C11	2.70 (13)	O1—C11—C12—C21	11.11 (17)
C1—C2—C3—C11	−178.96 (9)	C3—C11—C12—C21	−171.67 (10)
C2—C3—C4—C5	−0.43 (15)	C21—C12—C13—C14	−0.74 (16)
C11—C3—C4—C5	179.59 (9)	C11—C12—C13—C14	177.45 (9)
C3—C4—C5—C10	−0.18 (15)	C12—C13—C14—C15	−178.53 (9)
C3—C4—C5—C6	179.70 (10)	C12—C13—C14—C19	1.56 (15)
C4—C5—C6—C7	−179.03 (11)	C13—C14—C15—C16	−179.92 (9)
C10—C5—C6—C7	0.85 (18)	C19—C14—C15—C16	−0.01 (15)
C5—C6—C7—C8	−0.7 (2)	C14—C15—C16—C17	0.94 (16)
C23—O3—C8—C9	−0.16 (16)	C15—C16—C17—C18	−0.79 (18)
C23—O3—C8—C7	179.06 (11)	C16—C17—C18—C19	−0.3 (2)
C6—C7—C8—C9	0.08 (19)	C17—C18—C19—C20	−178.80 (13)
C6—C7—C8—O3	−179.18 (11)	C17—C18—C19—C14	1.23 (19)
O3—C8—C9—C10	179.53 (10)	C13—C14—C19—C18	178.85 (10)
C7—C8—C9—C10	0.36 (17)	C15—C14—C19—C18	−1.07 (16)
C4—C5—C10—C1	0.18 (14)	C13—C14—C19—C20	−1.11 (16)
C6—C5—C10—C1	−179.70 (10)	C15—C14—C19—C20	178.97 (11)
C4—C5—C10—C9	179.48 (9)	C18—C19—C20—C21	179.91 (14)
C6—C5—C10—C9	−0.40 (15)	C14—C19—C20—C21	−0.1 (2)
C2—C1—C10—C5	0.43 (15)	C19—C20—C21—C12	1.0 (2)
C2—C1—C10—C9	−178.86 (9)	C13—C12—C21—C20	−0.54 (19)
C8—C9—C10—C5	−0.18 (15)	C11—C12—C21—C20	−178.75 (12)
C8—C9—C10—C1	179.10 (9)		

### Hydrogen-bond geometry (Å, º)

Cg1 is the centroid of the C14–C19 ring.

**Table:**

*D*—H···*A*	*D*—H	H···*A*	*D*···*A*	*D*—H···*A*
C22—H22*A*···*Cg*4^i^	0.98	2.80	3.5470 (12)	133

Symmetry code: (i) *x*, *y*−1, *z*.
